# Isolation of osteogenic progenitors from human amniotic fluid using a single step culture protocol

**DOI:** 10.1186/1472-6750-9-9

**Published:** 2009-02-16

**Authors:** Ivana Antonucci, Irene Iezzi, Elisena Morizio, Filiberto Mastrangelo, Andrea Pantalone, Monica Mattioli-Belmonte, Antonio Gigante, Vincenzo Salini, Giuseppe Calabrese, Stefano Tetè, Giandomenico Palka, Liborio Stuppia

**Affiliations:** 1 Department of Biomedical Sciences, "G. d'Annunzio" University, Chieti-Pescara, Italy; 2 Aging Research Center (CE.S.I.), "G. d'Annunzio" University Foundation, Chieti-Pescara, Italy; 3 Human Genetics Division, Pescara Hospital, Pescara, Italy; 4 Department of Oral Sciences "G. d'Annunzio" University, Chieti-Pescara, Italy; 5 Orthopedic and Traumatologic Division, "G. d'Annunzio" University, Chieti-Pescara, Italy; 6 Department of Molecular Pathology and Innovative Therapies, Polytechnic University of Marche, Ancona, Italy; 7 Institute for Molecular Genetics, National Research Council (CNR), Bologna, Italy

## Abstract

**Background:**

Stem cells isolated from amniotic fluid are known to be able to differentiate into different cells types, being thus considered as a potential tool for cellular therapy of different human diseases. In the present study, we report a novel single step protocol for the osteoblastic differentiation of human amniotic fluid cells.

**Results:**

The described protocol is able to provide osteoblastic cells producing nodules of calcium mineralization within 18 days from withdrawal of amniotic fluid samples. These cells display a complete expression of osteogenic markers (COL1, ONC, OPN, OCN, OPG, BSP, Runx2) within 30 days from withdrawal. In order to test the ability of these cells to proliferate on surfaces commonly used in oral osteointegrated implantology, we carried out cultures onto different test disks, namely smooth copper, machined titanium and Sandblasted and Acid Etching titanium (SLA titanium). Electron microscopy analysis evidenced the best cell growth on this latter surface.

**Conclusion:**

The described protocol provides an efficient and time-saving tool for the production of osteogenic cells from amniotic fluid that in the future could be used in oral osteointegrated implantology.

## Background

Amniotic Fluid Cells (AFCs) can be classified in epitheloid E-type cells, amniotic fluid specific AF-type cells and fibroblastic F-type cells (1). In recent years, different reports have demonstrated that presence in human amniotic fluid of stem cells (AFS) able to differentiate into multiple lineages [1-8]. Very recently, the ability of clonal AFS to produce cell types inclusive of all embryonic germ layers was demonstrated [9,10]. Unlike embryonic stem cells, AFS have been showed to be not tumorigenic after transplantation in mice [9]. As a consequence, several studies have suggested the usefulness of these cells for therapeutic purposes [11-16]. Osteoblastic cells derived from AFS could be useful for bone regeneration after traumatic or degenerative damage [17,18]. In fact, osteoblastic progenitors obtained from amniotic fluid could be used to engineer the craniofacial structures whose natural development is regulated by mesenchymal cells originating from the neural crest, avoiding long and difficult therapies of bone augmentation with intra-oral or extra oral donor site [19,20]. In order to obtain the best results in craniofacial tissue engineering, great relevance is assumed by the use of scaffolds able to accommodate cell growth and tissue genesis. To date, implants with different surface treatments are investigated to define the best surface morphology for a good osteoblastic cell proliferation and osseointegration around implant [21-25].

The aim of the present study is to evaluate the ability of human AFS to differentiate into osteogenic cells using a novel single step culture protocol, and to test their growth ability on different implant surfaces.

## Results

Osteoblastic differentiation was obtained in the present study using two different culture protocols of amniotic fluid cells. In the first protocol (Protocol 1), Amniotic Fluid Mesenchymal Stem cells (AFMSCs) were transferred in osteogenic medium at passage 6, while in the second protocol (Protocol 2), pellets of amniotic fluid samples were directly resuspended in osteogenic medium without the selection of AFMSCs.

**Figure 1:**
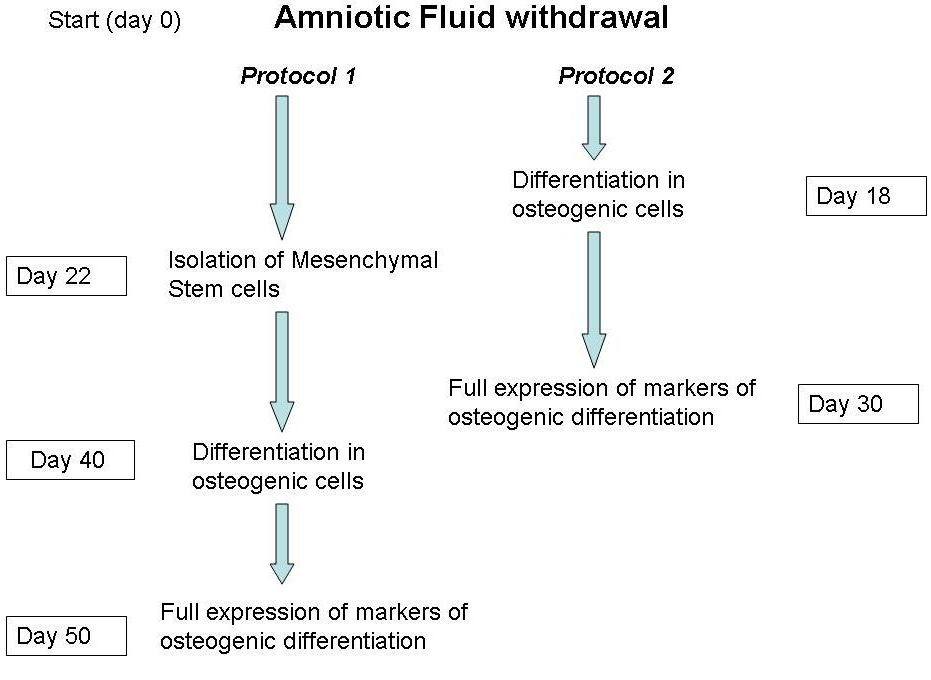
**Flow chart showing the different steps of the two protocols for the production of osteogenic cells from amniotic fluid**.

A flow chart describing the different timing of the two protocols used in this study is reported in Figure 1.

**Figure 2:**
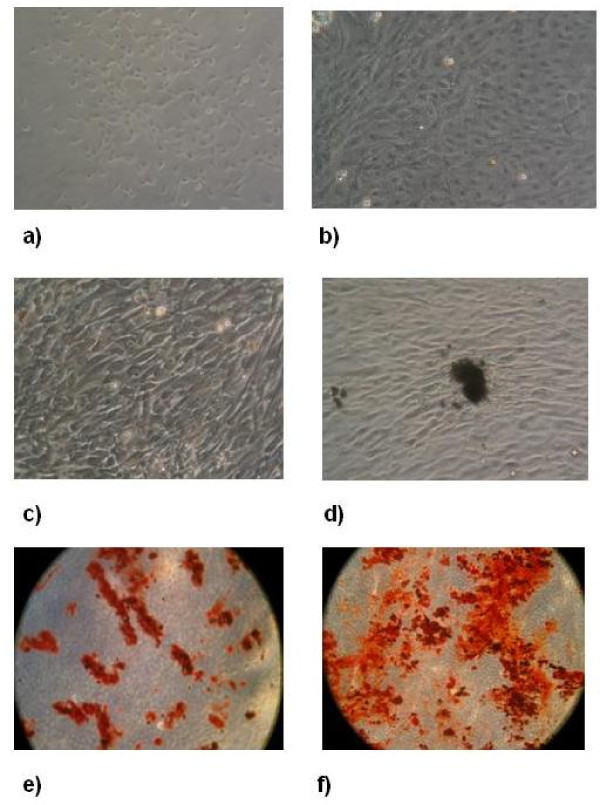
**a) Fibroblast-like cells (AFMSCs) obtained after 7 days of amniotic fluid culture (Protocol 1); b) Confluence of AFMSCs after 22 days of amniotic fluid culture (Protocol 1); c) over confluent osteoblastic cells after 20 days of amniotic fluid culture in osteogenic medium (Protocol 2); d) nodules of calcium mineralization, osteoblastic cells (Protocol 2); e) Alizarin Red Staining of osteoblastic cells obtained after 22 days of amniotic fluid culture**. Red spots indicate the presence of calcium mineralization; f) Alizarin Red Staining of osteoblastic cells after 30 days of amniotic fluid culture. Note the increase in the number and size of aggregates of calcium mineralization.

**Figure 3:**
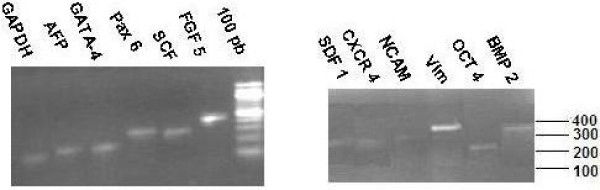
**RT-PCR analysis of AFMSCs at day 20 of culture (protocol 1)**.

In Protocol 1, seven days after the initiation of the primary culture, fibroblast-like cells appeared both isolated and as colonies in the culture flask (Figure 2a). After 20–22 days of culture, at 70–80% confluence (Figure 2b), cells were treated with trypsin and EDTA and collected. RT-PCR analysis, carried out on RNA extracted from the cells at this stage, evidenced the presence of genes previously reported as expressed in AFMSCs [26], namely SDF1, CXCR4, Oct-4, SCF, GATA-4, Vim, FGF-5, Pax-6, NCAM, AFP, BMP-2 (Figure 3). Cells collected at day 20–22 were transferred and cultured in the osteogenic medium. After 18 days of culture in osteogenic medium (day 40 from withdrawal), the cells showed 70–75% confluence, and the presence of aggregates or nodules of calcium mineralization was appreciable. The number and size of these aggregates increased in the following days. Cells directly cultured in osteogenic medium (Protocol 2) reached 70–75% confluence after 18 days from withdrawal, and became over confluent in the following days (Figure 2c). In the following days the appearance of the first aggregates of calcium mineralization was observed (Figure 2). Alizarin Red staining confirmed the presence of biomineralization (Fig. 2e). An increase in the number and size of aggregates during the time was observed also in these cultures (Figure 2f). Cell count carried out on 5 cultures performed with protocol 2 at day 30 from withdrawal demonstrated the presence of cell number ranging from 8,9 × 10^6 ^9,7 × 10^6 ^cells.

**Figure 4:**
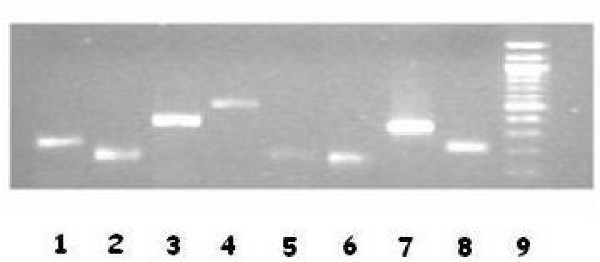
**RT-PCR analysis of osteoblastic cells at 30 days of culture (protocol 2)**. Line 1 = ONC; Line 2 = Runx2; Line 3 = OCN; Line 4 = BSP; Line 5 = OPN; Line 6 = COL I; Line 7 = OPG; Line 8 = GAPDH; Line 9 = 100 bp molecular weight marker.

RT-PCR analysis carried out at day 50 (protocol 1) or 30 (Protocol 2) from withdrawal, showed expression of COL1, ONC, OPN, OCN, OPG, BSP and Runx2, typical markers of the osteogenic differentiation (Figure 4). The same genes were not expressed in fresh amniotic fluid samples, analyzed as negative control (not showed).

**Figure 5:**
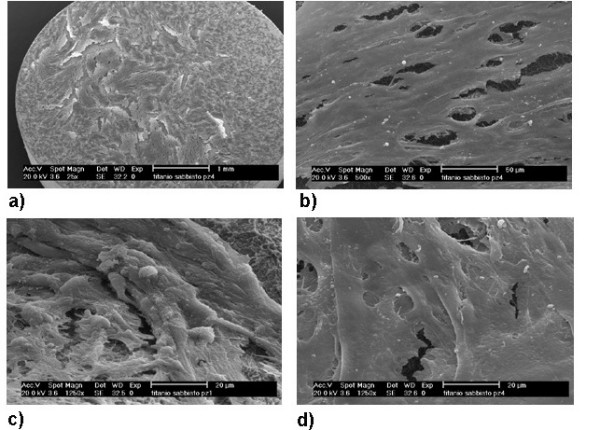
**Scanning Electron Microscope analysis of osteoblastic cells cultured on SLA titanium disks**. a) 22×, b) 500×: adherent cells covering the whole surface of SLA titanium disk; c) 1250×, d) 1250×: evidence of philophodia surrounding cell surfaces.

In order to evaluate the growth ability of osteoblastic cells obtained by Protocol 2 on different surfaces commonly used in oral implantology, cultures were carried out on smooth copper, machined titanium and Sandblasted and Acid Etching titanium (SLA titanium) test disks, and evaluated using Electron Scanning Microscopy. Titanium is universally considered as the first-rate material for oral osseointegrated implantology. Additional treatments on commercially pure (c.p.) titanium surface provide further enhancement of bone-to-implant contact, thus reducing the osseointegration period, improving treatment outcome and increasing applicability to poor bone quality. The investigation of implants with different surface treatments, both in vitro and in vivo, is a crucial point in order to define the surface morphology which could permit a good osteoblastic cell proliferation and osseointegration around implant. In our experiments, adherent cells were not detected on smooth copper surface (negative control) at day 3, while different behaviour of osteoblastic-like cells were observed on machined titanium and SLA titanium surfaces. On machined titanium surfaces, few adherent cells were observed around the titanium disk. On the contrary, adherent cells were found to cover the whole surface of SLA titanium disk (Figure 5a). Cell aggregates were arranged almost uniformly and formed a single layer cell culture on the disk surface (Figure 5b). At high magnification philophodia surrounding cell surfaces were clearly visible (Figure 5c–d).

In order to evaluate the mitotic stability of cells, cytogenetic investigation was carried out on Protocol 2 cultures at day 30, showing normal diploid karyotype in all the investigated metaphases.

## Discussion

Different protocols have been reported in literature for the differentiation of osteogenic cells starting from amniotic fluid. Some authors reported the use of immunoselection with c-Kit specific antibodies in order to isolate AFS starting from confluent human amniocentesis cultures, followed by proliferation of AFMSCs under appropriate culture conditions, and finally osteoblastic differentiation after several days of culture [8,9]. On the other hand, other groups cultured unselected amniotic fluid cells in media allowing the proliferation of AFMSCs, and subsequently induced their differentiation in osteoblastic cells [1,4,5].

In the present study, we demonstrated the ability of human AFS to differentiate into osteogenic cells using a single step culture procedure, allowing a 20 days reduction of the culture time as compared to previously reported protocols. This could represent an important point in the view of a possible therapeutic application of these cells. Amniotic fluid samples, directly resuspended in osteogenic medium without the selection of AFMSCs, were able to produce osteogenic cells after 18 days from the withdrawal as demonstrated by Alizarin Red staining. RT-PCR analysis showed the full expression of all osteogenic markers typical of late stage osteoblasts after 30 days of colture, while the same expression pattern is showed after 50 days from withdrawal by cells obtained using conventional protocols. Cytogenetic investigation, carried out at day 30 on cells obtained by the single step protocol, showed normal diploid karyotype in all the investigated samples, thus confirming the mitotic stability of cells obtained using this procedure. Cell count performed on cultures carried out with protocol 2 showed the presence at day 30 from withdrawal of about 9,7 × 10^6 ^cells starting from as little as 2–3 ml of amniotic fluid. Although the direct culture of AFS cells in osteogenic medium likely induces a complete cell differentiation within 30 days, with arrest of cell proliferation, the amount of cells obtained with this protocol fits well with the cell number required for preclinical studies in animal models and for local transplant in human. Since this latter approach would likely represent the gold standard for a future clinical application in odontoiatric and orthopaedic implantology, the cell number obtained using our direct protocol appears to be sufficient for future local therapeutic purposes.

In order to test the ability of osteoblastic cells obtained from amniotic fluid to proliferate onto surfaces commonly used for craniofacial implantology, and to evaluate their usefulness for tissue engineering, we tested these cells on disks with machined titanium and SLA titanium surfaces. Electron microscopy observation showed a good growth and adherence of osteoblastic cells on this latter surface. This result indicates the excellent biocompatibility of osteoblastic cells obtained from amniotic fluid with SLA titanium scaffolds currently utilized in dental implant.

## Conclusion

The protocol described in the present study shows the ability of producing osteoblastic cells from amniotic fluid samples in a very short time, being these cells fully differentiated within one month from withdrawal. Although osteoblastic progenitors can be successfully obtained from bone marrow stromal cells, the use of amniotic fluid as a source of these cells is of relevance since AFS can be easily obtained from routine clinical amniocentesis specimens that would otherwise be discarded. Thus, it is possible to suggest that banking of these stem cells will provide in the future a relevant source both for autologous therapy in the adulthood and for the transplant in HLA matched recipients.

## Methods

### Isolation and culture of mesenchymal stem cells from amniotic fluid (AFMSCs)

Amniotic fluid samples were obtained from 11 women undergoing amniocentesis for prenatal diagnosis at 16–19 weeks of pregnancy after written informed consent. The study has been approved by the Ethics Committee for Biomedical Research of the "G. d'Annunzio" University, Chieti. For each sample, 2–3 ml of amniotic fluid, corresponding to a cell number ranging from 2 × 10^3 ^to 2 × 10^6 ^[1] were centrifuged for 10 minutes at 1800 rpm. Pellets were resuspended in Iscove's modified Dulbecco's medium supplemented with 20% FBS, 100 U/ml penicillin, 100 μg/ml streptomycin (Sigma), 2 mM L-glutamine, 5 ng/ml basic fibroblast growth factor (FGF2) and incubated at 37°C with 5% humidified CO2. After 7 days, non-adherent cells were removed and the adherent cells allowed to growth in the same medium, which was changed each 4 days. When culture reached confluence (about 20 days after the primary culture), cells were treated with 0,05% trypsin and 0,02% EDTA, then counted and replaced in 25 cm^2 ^culture flasks.

### Osteogenic differentiation

Two different culture protocols were used for osteogenic differentiation of amniotic fluid cells. In the first protocol (Protocol 1), AFMSCs cells at passage 6 were transferred in osteogenic medium consisting of the above described medium with the addition of 150 μg/ml β Glycerophosphate, 50 μg/ml ascorbic acid, and 10^-8 ^M dexamethasone. In the second protocol (Protocol 2), pellets of amniotic fluid samples were directly resuspended in osteogenic medium in 75 cm^2 ^flasks without the selection of AFMSCs. At day 8 from withdrawal, colony forming cells were counted, showing a number ranging from 20 to 20.000 in the different cultures. To visualize calcium sediments, cells treated with Protocol 2 were stained at different times (19, 22 and 30 days from withdrawal) with Alizarin Red S solution, according to Gregory et al. [27]. Mineralization was demonstrated by the presence of red depositions. All reagents used for cells culture and staining were purchased by Sigma-Aldrich (Milano, Italy)

### Culture on different surfaces

Three test disks (diameter 10 mm, thickness 5 mm) for each different surface, namely smooth copper, machined titanium and Sandblasted and Acid Etching titanium (SLA titanium), were used in this study Geometric surface morphology of Machined Titanium test disks was obtained with turning machined treatment with formation of titanium micro-parallel walls. Sandblasted and Acid Etching titanium (SLA titanium) test disks were obtained by TiO2 particles being applied to the surface and two phases of etching with fluoridric acid followed by a second acid attack by sulphuric-hydrochloric acid with irregular distributed porosity structure of micro-deep valleys alternated to elevated sharp crests. To preliminary characterize the surface morphology, test disks have been evaluated by means of Scanning Electron Microscopy (SEM) imaging (LEO 435 VP, Cambridge, UK) at about 15–20 kV, high vacuum mode. The surface roughness of the specimens were measured with a stylus profilometer (ANSI/ASME B46.1 1–2002) and a gloss meter (45°-90° sensor angle, 1–10 range, DIN 16537). Differences between treatment groups were evaluated using an analysis of variance at the 95% confidence level and parametric Newman-Keuls multiple comparison test at p = 0.05 significance level. After differentiation, at day 15, osteoblastic cells obtained using protocol 2 were divided in three groups and 3,7 × 10^4 ^cells were seeded onto each of the three different test disks. When 70% confluence was observed (after 2–3 days of culture), cells were prepared and analysed by SEM. The entire culture protocol on test disks was repeated two times.

### Scanning Electron microscopy

For SEM analysis, specimens cells were fixed in 2% gluteraldehyde in 0.1 M cacodylate buffer (pH 7.4). To preserve the lipid structures, specimens were gently washed in 0.2 M cacodylate buffer (pH 7.4) with the addition of 0.15 M saccharose for three changes every 20 minutes, post-fixed in 1% osmium tetroxide at room temperature for 1 hours, then given two quick changes of the previous buffer and gradually dehydrated in increasing ethanol concentrations (from 25 to 100%, 15% steps). Samples were then carried through critical point drying (CPD) according to standard procedure using liquid carbon dioxide, mounted on aluminium stubs, gold-sputtered and observed with a Philips XL20 Scanning Electron Microscope (SEM Philips XL 20; FEI, Eindhoven, The Netherlands) at 20 KV, high vacuum mode. Images were stored in TIF format with 1024 × 768 Grid of Pixels

### RT-PCR

Total RNA was isolated using the SV Total RNA Isolation System Kit (Promega, Milano, Italy) from: a) AFMSCs cells after 20 days culture in standard medium (protocol 1); b) differentiated cells after 30 days in osteogenic medium (protocols 1 and 2). RNA from fresh amniotic fluid was also used as a control. One μg of total RNA was reverse transcribed using RETROscript Kit (Ambion, Milano, Italy).

Amplification was performed with specific primers for two classes of genes (table. 1): a) genes expressed in mesenchymal cells (SDF1, CXCR4, Oct-4, SCF, GATA-4, Vim, FGF-5, Pax-6, NCAM, AFP, BMP-2) (26, 28); b) genes expressed during osteogenic differentiation (COL1, ONC, OPN, OCN, OPG, BSP and Runx2) [29-31]. Amplifications were carried out using 35 cycles of 95°C, 1 min; variable annealing temperature (see Table 1), 1 min; 72°C, 1 min. RT-PCR products were separated in a 2% agarose gel and visualized by Ethidium Bromide staining. Images were captured using a Gel Doc 2000 (BioRad, CA, USA).

**Table 1:** Genes analyzed in RT-PCR experiments, primer sequences and annealing temperature.

**Gene**	**Gene symbol**	**Primer Sequences**	**Annealing temperature**	**Size (bp)**
Stromal cell-derived factor-1	SDF1	F – gacccgcgctcgtccgcc R – cgggtcaatgcacacacttgtcta	57°	262

Chemokine (C-X-C motif) receptor 4	CXCR4	F – agctgttggctgaaaaggtgg R – gcgcttctggtggcccttgga	60°	260

Octamer-binding transcription factor 4	Oct-4	F – cgt gaa gct gga gaa gga gaa gct g R – caa ggg ccg cag ctc aca cat gtt c	60°	245

Stem cell factor	SCF	F – cca ttg atg cct tca agg ac R – ctt cca gta taa ggc tcc aa	62°	275

GATA binding protein 4	GATA-4	F – ttc ctc ttc cct cct caa at R – tca gcg tgt aaa ggc atc tg	60°	194

Vimentin	Vim	F – tca gcg tgt aaa ggc atc tg R – cct tcg tga ata cca cg acct gc	56°	321

Fibroblast growth factor 5	FGF-5	F – gct gtg tct cag ggg att gta gga ata R – tat cca aag cga aac ttg agt ctg ta	62°	434

Paired box 6	Pax-6	F – aga ttc aga tga ggc tca aa R – aat tgg ttg gta gac act gg	60°	313

Neural cell adhesion molecule	NCAM	F – gag ggg gaa gat gcc gtg atg tg R – ata ttc tgc ctg gcc cgg atg gta g	63°	269

Bone morphogenetic protein 2	BMP-2	F – ttg cgg ctg ctc agc atg tt R – ttg cga gaa cag atg caa gat g	62°	315

Alpha-fetoprotein	AFP	F – gtg ctg cac ttc ttc ata tgc R – tga cag cct caa gtt gtt cc	60°	218

Type I collagen	COL1	F – ttcctttgcattcatctctca R – caagtggaccaagcttcctt	58°	149

Osteonectin	ONC	F – gtctcactggctgtgttgga R – aagacttgccatgtgggttc	60°	215

Osteopontin	OPN	F – aggaggaggcagagcaca R – ctggtatggcacaggtgatg	60°	152

Osteocalcin	OCN	F – catgagagccctcaca R – agagcgacaccctagac	58°	315

Osteoprotegerin	OPG	F – tgctgttcctacaaagttttacg R – ctttgagtgctttagtgcgtg	60°	433

Bone sialoprotein	BSP	F – ctatggaaggacgccacgcct R – catagccatcgtagccttgtcc	62°	578

Runt-related transcription factor 2	Runx2	F – gacagaagcttgatgactctaaacc R – tctgtaatctgactctgtccttgt	60°	169

Glyceraldehyde-3-phosphate dehydrogenase	GAPDH	F – ccatggagaaggctggg R – caaagttgtcatggatgacc	60°	194

### Cytogenetic investigation

For cytogenetic analysis, cultures carried out using protocol 2 were treated at day 30 with trypsin and 36–48 hours colcemid. Metaphase chromosomes were stained with GTG-banding and Giemsa. At least 20 metaphases were examined for each sample.

## Authors' contributions

IA carried out cell cultures and osteoblastic differentiation, performed molecular genetics experiments, partecipated to the design of the study and to the drafting of the manuscript. IE carried out cytogenetic investigation. EM partecipated to AF cultures. FM partecipated to AF cells cultures on sample disks. AP prepared osteoblastic cells for SEM analysis. MMB partecipated in osteoblastic cells analysis by SEM. AG carried out osteoblastic cells analysis by SEM. VS partecipated in the design of the study. GC provided human AF samples. ST partecipated to the design of the study and to the drafting of the manuscrupt. GP partecipated in the design of the study and performed genetic counselling on women undergoing amniocentesis. LS coordinated the study and partecipated to the drafting of the manuscrupt. All authors read and approved the final manuscript.
